# A six-microRNA panel in plasma was identified as a potential biomarker for lung adenocarcinoma diagnosis

**DOI:** 10.18632/oncotarget.14311

**Published:** 2016-12-27

**Authors:** Xin Zhou, Wei Wen, Xia Shan, Wei Zhu, Jing Xu, Renhua Guo, Wenfang Cheng, Fang Wang, Lian-Wen Qi, Yan Chen, Zebo Huang, Tongshan Wang, Danxia Zhu, Ping Liu, Yongqian Shu

**Affiliations:** ^1^ Department of Oncology, First Affiliated Hospital of Nanjing Medical University, Nanjing 210029, PR China; ^2^ Department of Thoracic Surgery, First Affiliated Hospital of Nanjing Medical University, Nanjing 210029, PR China; ^3^ Department of Respiration, The Affiliated Jiangning Hospital of Nanjing Medical University, Nanjing 210000, PR China; ^4^ Department of Gastroenterology, First Affiliated Hospital of Nanjing Medical University, Nanjing 210029, PR China; ^5^ Department of Cardiology, First Affiliated Hospital of Nanjing Medical University,Nanjing 210029, PR China; ^6^ State Key Laboratory of Natural Medicines and Department of Pharmacognosy, China Pharmaceutical University, Nanjing, 210009, China; ^7^ Department of Emergency, First Affiliated Hospital of Nanjing Medical University, Nanjing 210029, PR China; ^8^ Department of Oncology, The Third Affiliated Hospital of Soochow University, Changzhou 213003, China; ^9^ Cancer Center of Nanjing Medical University, Nanjing 210029, China

**Keywords:** plasma, miRNA, lung adenocarcinoma, diagnosis, exosomes

## Abstract

Differently expressed microRNAs (miRNAs) in the plasma of lung adenocarcinoma (LA) patients might serve as biomarkers for LA detection. MiRNA expression profiling was performed using Exiqon panels followed by the verification (30 LA VS. 10 healthy controls (HCs)) with quantitative reverse transcription polymerase chain reaction (qRT-PCR) in the screening phase. Identified miRNAs were confirmed through training (42 LA VS. 32 HCs) and testing stages (66 LA VS. 62 HCs) by using qRT-PCR based absolute quantification methods. A total of six up-regulated plasma miRNAs (miR-19b-3p, miR-21-5p, miR-221-3p, miR-409-3p, miR-425-5p and miR-584-5p) were identified. The six-miRNA panel could discriminate LA patients from HCs with areas under the receiver operating characteristic curve of 0.72, 0.74 and 0.84 for the training, testing and the external validation stage (33 LA VS. 30 HCs), respectively. All the miRNAs identified except miR-584-5p were significantly up-regulated in LA tissues. MiR-19-3p, miR-21-5p, miR-409-3p and miR-425-5p showed high expression in arterial plasma with borderline significance. Additionally, miR-19-3p, miR-21-5p and miR-221-3p were significantly up-regulated in exosomes extracted from LA peripheral plasma samples. In conclusion, we identified a six-miRNA panel in peripheral plasma which might give assistance to the detection of LA at least for Asian population to a certain extent.

## INTRODUCTION

Lung cancer is one of the most fatal malignancies and the leading cause of cancer-related death worldwide [1]. Occupying more than 80% of lung cancer, non-small cell lung cancer (NSCLC) includes squamous cell carcinoma (SCC), large cell carcinoma and adenocarcinoma (LA) [2, 3]. Surgical resection remains to be the most effective treatment for NSCLC at present. However, due to lack of apparent symptoms, many patients are diagnosed with locally advanced and advanced stages and do not have a choice of surgery which leads to a low 5-year overall survival rate (less than 15%) for NSCLC patients. Despite increased understanding of the molecular and clinical characteristics of NSCLC as well as recent advances in screening and treatment strategies [4–7], the prognosis of NSCLC is still poor. Computed tomography (CT) especially low-dose CT is currently used as the screening method for NSCLC. But potential over-diagnosis and harmful effects induced by radiation limit their use in the clinical [8]. Existing protein biomarkers such as carcinoembryonic antigen (CEA) and CYFRA21-1 did not show sufficient sensitivity and specificity [9, 10]. In view of this, novel and reliable biomarkers to detect NSCLC for early intervention with the potential to reduce mortality are urgently needed.

MicroRNAs (miRNAs) are short (typically 18-25 nucleotides), single-stranded and highly conserved non-coding RNAs which could negatively regulate gene expression at post-transcriptional level by binding the 3′-untranslated region of target mRNAs, resulting in either mRNA degradation or translational repression [11, 12]. Passively leaked or actively transported from cells, circulating miRNAs could be stably detected in blood and have been used as biomarkers for diagnosis, prognosis or monitoring curative effect in various cancers including NSCLC [13]. Most previous studies focused on the differential expression levels of miRNAs in patients with NSCLC, however, the results were not consistent due to diverse research methods and tested populations [14]. Different subtypes of NSCLC showed different molecular alterations, biological behaviors and might also own different miRNA profilings. Recent clinical trials have shown that sub-grouping NSCLC into subtypes could achieve maximum benefit while minimising toxicity for patients [15, 16]. Comprehensive molecular profiling of LA (the largest subgroup of NSCLC) revealed by The Cancer Genome Atlas (TCGA) project could also establish a foundation for classification of NSCLC [17]. Thus, there is an urgent need in considering subtypes when aiming to identify biomarkers. In addition, differences of preanalytical and analytical variables could impact the accuracy of detecting circulating miRNAs [18]. However, absolute quantification procedure based on the spiked-in normalization method seems to be the optimal way to normalize miRNA between body fluid samples [19–21].

In the present study, we focused on the specific subtype of NSCLC (LA) and conducted a four-phase study to identify potential miRNAs for detecting LA by evaluating the absolute concentration of plasma miRNAs based on quantitative reverse transcription polymerase chain reaction (qRT-PCR). In addition, the identified miRNAs were confirmed in tissue samples and compared in arterial and peripheral plasma samples. Exosomal miRNAs were also investigated to assess the potential form of the identified miRNAs in peripheral plasma which might aid detection of LA.

## RESULTS

### Characteristics of subjects

**Figure 1:**
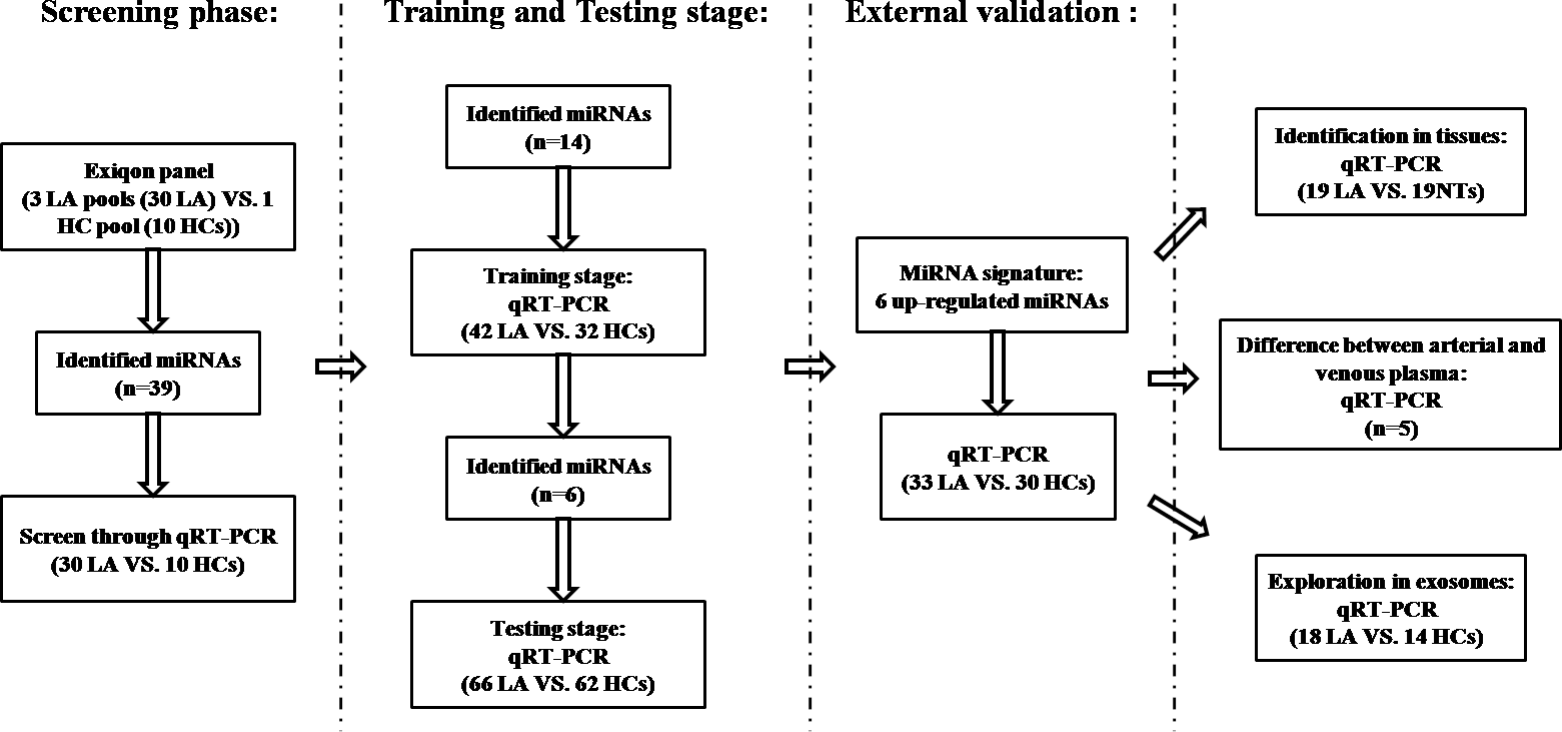
The flow chart of the experiment design LA: lung adenocarcinoma; HC: healthy control; NT: normal tissue.

A total of 265 subjects, including 141 LA patients and 124 healthy controls (HCs), were included in our study. The flow chart of the experiment (Figure 1) showed that our study was divided into three stages after the screening phase: the training stage, the testing stage, and the external validation stage. Each pooled sample analyzed in the screening phase was randomly selected from the subjects, and per 10 samples were pooled as 1 pool sample. As shown in Table 1, there was no significant difference in the distribution of age or gender between LA patients and HCs in any stage (*p*-values > 0.05).

**Table 1:** Characteristics of 141 LA patients and 124 healthy controls included in the study

	Screening phase (*n* = 40)		Training cohort (*n* = 74)		Testing cohort (*n* = 128)		External validation cohort (*n* = 63)	
Variables	Cases (%)	Controls (%)	Cases (%)	Controls (%)	Cases (%)	Controls (%)	Cases (%)	Controls (%)
**Number**	30	10	42	32	66	62	33	30
**Gender**								
Male	14 (46.7)	4 (40)	16 (38.1)	15 (46.9)	30 (45.4)	28 (45.2)	15 (45.5)	14 (46.7)
Female	16 (53.3)	6 (60)	26 (61.9)	17 (53.1)	36 (54.6)	34 (54.8)	18 (54.5)	16 (53.3)
**Age**								
< 60	12 (40)	5 (50)	24 (57.1)	20 (62.5)	28 (42.4)	30 (48.4)	16 (48.5)	13 (43.3)
≥ 60	18 (60)	5 (50)	18 (42.9)	12 (37.5)	38 (57.6)	32 (51.6)	17 (51.5)	17 (56.7)
**Smoking**								
Smoker	2 (6.7)		2 (4.8)		5 (7.6)		3 (9.1)	
Non-smoker	21 (70)		37 (88.1)		57 (86.4)		28 (84.9)	
NA	7 (23.3)		3 (7.1)		4 (6)		2 (6)	
**TNM stage**								
I	9 (30)		14 (33.3)		18 (27.3)		9 (27.3)	
II	5 (16.7)		9 (21.4)		15 (22.7)		8 (24.2)	
III	5 (16.7)		10 (23.9)		19 (28.7)		10 (30.3)	
IV	11 (36.6)		9 (21.4)		14 (21.3)		6 (18.2)	
**EGFR mutation**								
Yes	5 (16.7)		8 (19.1)		14 (21.2)		4 (12.1)	
No	6 (20)		7 (16.7)		8 (12.1)		5 (15.2)	
NA	19 (63.3)		27 (64.2)		44 (66.7)		24 (72.7)	

### MiRNA profiling from the screening phase

To identify differentially expressed miRNAs in peripheral plasma of LA patients, we initially screened 168 miRNAs by the Exiqon miRCURY-Ready-to-Use-PCR-Human-panel-I + II-V1.M on the qRT-PCR platform in 3 LA and 1 HC pooled samples. Only the miRNAs with cycle threshold (Ct) value < 37 and 5 lower than negative control in the panel were eligible for further analysis. A total of 39 miRNAs (36 up-regulated miRNAs and 3 down-regulated miRNAs; Supplementary Table S1 online) showed more than two-fold altered expression in all 3 pooled LA samples compared to the HC pool sample and were selected as candidate miRNAs. After that, to verify the reproducibility of the results from arrays and control the false discovery rate, the 39 miRNAs were confirmed by qRT-PCR in the same 40 participants. Fourteen miRNAs (Supplementary Table S1 online) including 13 up-regulated and 1 down-regulated miRNAs with mean fold change (FC) > 1.5 or < 0.66 and *P* value < 0.05 were identified and chosen to further validation stage outlined below.

### Validation of the miRNAs in peripheral plasma by qRT-PCR

**Figure 2:**
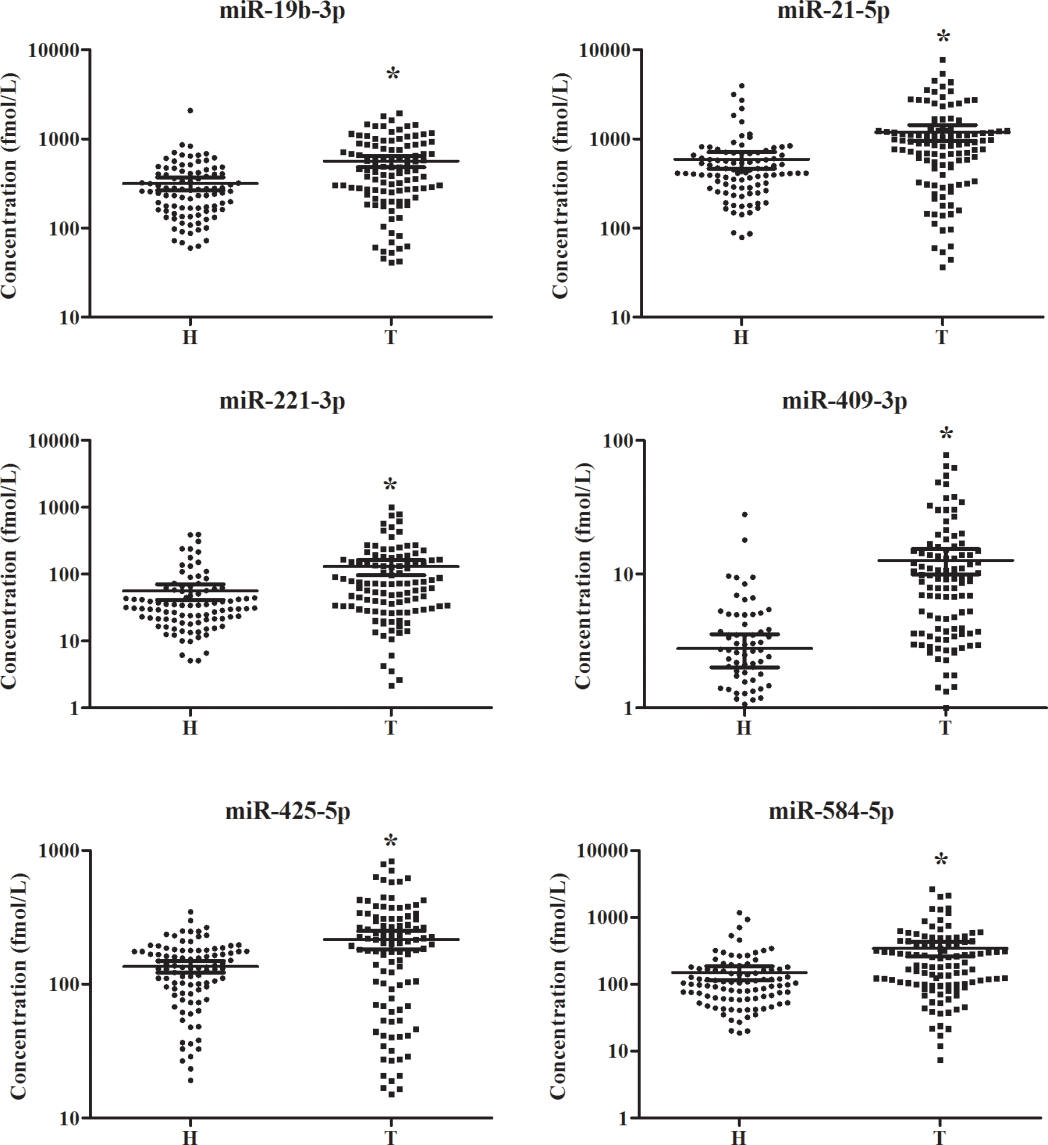
Expression levels of the six miRNAs in the peripheral plasma of 108 LA patients and 94 controls (in the training and testing stages) H: healthy controls; T: tumor. Horizontal line: mean with 95% CI. *P-value < 0.05.

The expression levels of the 14 miRNAs identified through the screening phase were evaluated in a larger sample set including 42 LA patients and 32 HCs in the training stage. Six miRNAs (miR-19b-3p, miR-21-5p, miR-221-3p, miR-409-3p, miR-425-5p and miR-584-5p) were found to be significantly up-regulated in plasma from LA patients (Table 2; the other 8 miRNAs were shown in the Supplementary Table S2). Next, the six miRNAs were subjected to validation in the testing stage with 66 LA patients and 62 HCs. And the differential expression patterns of the six miRNAs between LA patients and HCs were concordant between the training and the testing stage (Table 2). In addition, all the six miRNAs had significantly higher expression levels in peripheral plasma of LA patients as compared with HCs when the results of the two stages were combined (Table 2; Figure 2).

**Table 2:** Expression levels of the six miRNAs in the peripheral plasma in the training and testing stage (presented as mean ± SD; fmol/L)

miRNA	Training stage				Testing stage			Combined		
	Cases	Controls	FC	*P* value	Cases	Controls	FC	*P* value	FC	*P* value
miR-19b-3p	592 ± 458	331 ± 222	1.79	0.015	517 ± 447	313 ± 280	1.65	0.022	1.71	< 0.001
miR-21-5p	1338 ± 1567	632 ± 709	2.12	0.005	1103 ± 990	570 ± 546	1.93	< 0.001	2.02	< 0.001
miR-221-3p	127 ± 136	51 ± 56	2.49	0.003	132 ± 194	59 ± 81	2.23	0.001	2.31	< 0.001
miR-409-3p	14 ± 17	3.3 ± 5.5	4.26	0.005	12.3 ± 16	2.8 ± 2.4	4.45	0.033	4.39	< 0.001
miR-425-5p	262 ± 195	152 ± 69	1.72	0.017	201 ± 166	128 ± 64	1.57	0.015	1.64	< 0.001
miR-584-5p	330 ± 458	161 ± 197	2.05	0.007	353 ± 425	145 ± 164	2.43	< 0.001	2.28	< 0.001

FC: fold change.

### Diagnostic value of miRNAs in peripheral plasma

**Figure 3:**
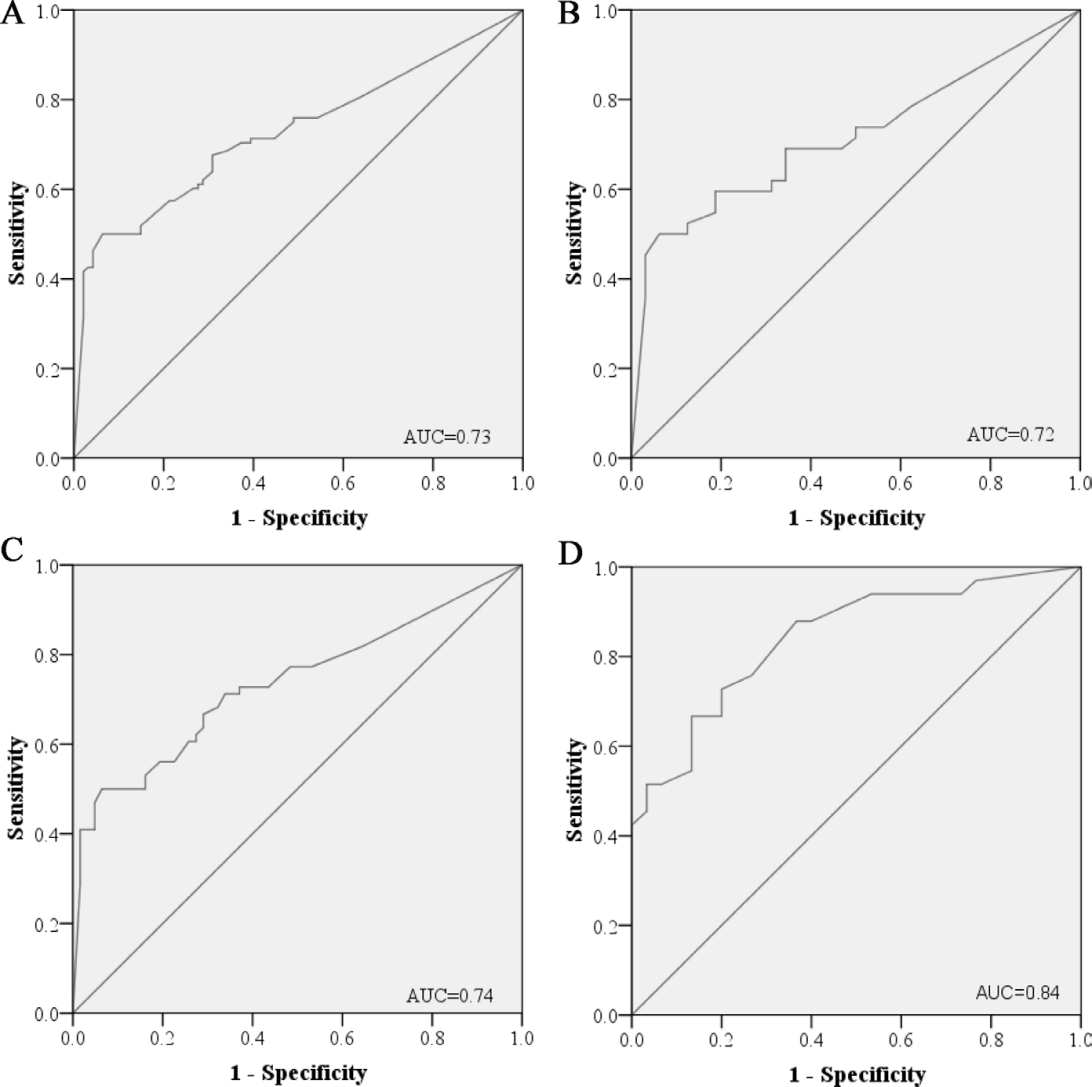
Receiver-operating characteristic (ROC) curves for the six-miRNA panel to discriminate LA patients from healthy controls (**A**) the combined two cohorts of training and testing stages (108 LA VS. 94 HCs); (**B**) training stage (42 LA VS. 32 HCs); (**C**) testing stage (66 LA VS. 62 HCs); (**D**) external validation stage (33 LA VS. 30 HCs). LA: lung adenocarcinoma; HC: healthy control. AUC: areas under the curve.

To evaluate the performance of the six miRNAs in discriminating LA patients from HCs, the optimal cutoff values for miR-19b-3p, miR-21-5p, miR-221-3p, miR-409-3p, miR-425-5p and miR-584-5p were determined according to ROC curves for each miRNA in the combined cohorts from the training and testing stage. AUCs for miR-19b-3p, miR-21-5p, miR-221-3p, miR-409-3p, miR-425-5p and miR-584-5p were 0.62 (95% confidence interval (CI): 0.54–0.7), 0.69 (95% CI: 0.62–0.77), 0.68 (95% CI: 0.61–0.76), 0.61 (95% CI: 0.53–0.69), 0.66 (95% CI: 0.58–0.74) and 0.69 (95% CI: 0.62–0.76), respectively (Supplementary Figure S1 online). To evaluate the diagnostic utility of the six-miRNA panel, the risk score function (RSF) of the six miRNAs for each subject was calculated. As shown in Figure 3A, the six-miRNA panel had a greater ability in distinguishing different groups than one particular miRNA in the combined cohorts with AUC of 0.73 (95% CI: 0.66–0.8; sensitivity = 68% and specifity = 70%). By using the same cutoff values, the diagnostic value of the six-miRNA panel was also assessed in the training and testing stage separately. The AUC of the panel was 0.72 (95% CI: 0.6–0.83; sensitivity = 69% and specifity = 66%) and 0.74 (95% CI: 0.65–0.82; sensitivity = 67% and specifity = 71%) for the training and the testing stage, respectively (Figure 3B and 3C).

To further confirm the diagnostic capacity of the six-miRNA panel for diagnosing LA, an additional cohort including 33 LA patients and 30 HCs was explored. Consistent with the results in the training and the testing stage, all six miRNAs showed significantly high expression level in plasma of LA patients (Supplementary Table S3 online). As shown in Figure 3D, the six-miRNA panel could accurately discriminate LA patients from HCs with AUC of 0.84 (95% CI: 0.75–0.94; sensitivity =73% and specifity = 80%).

**Figure 4:**
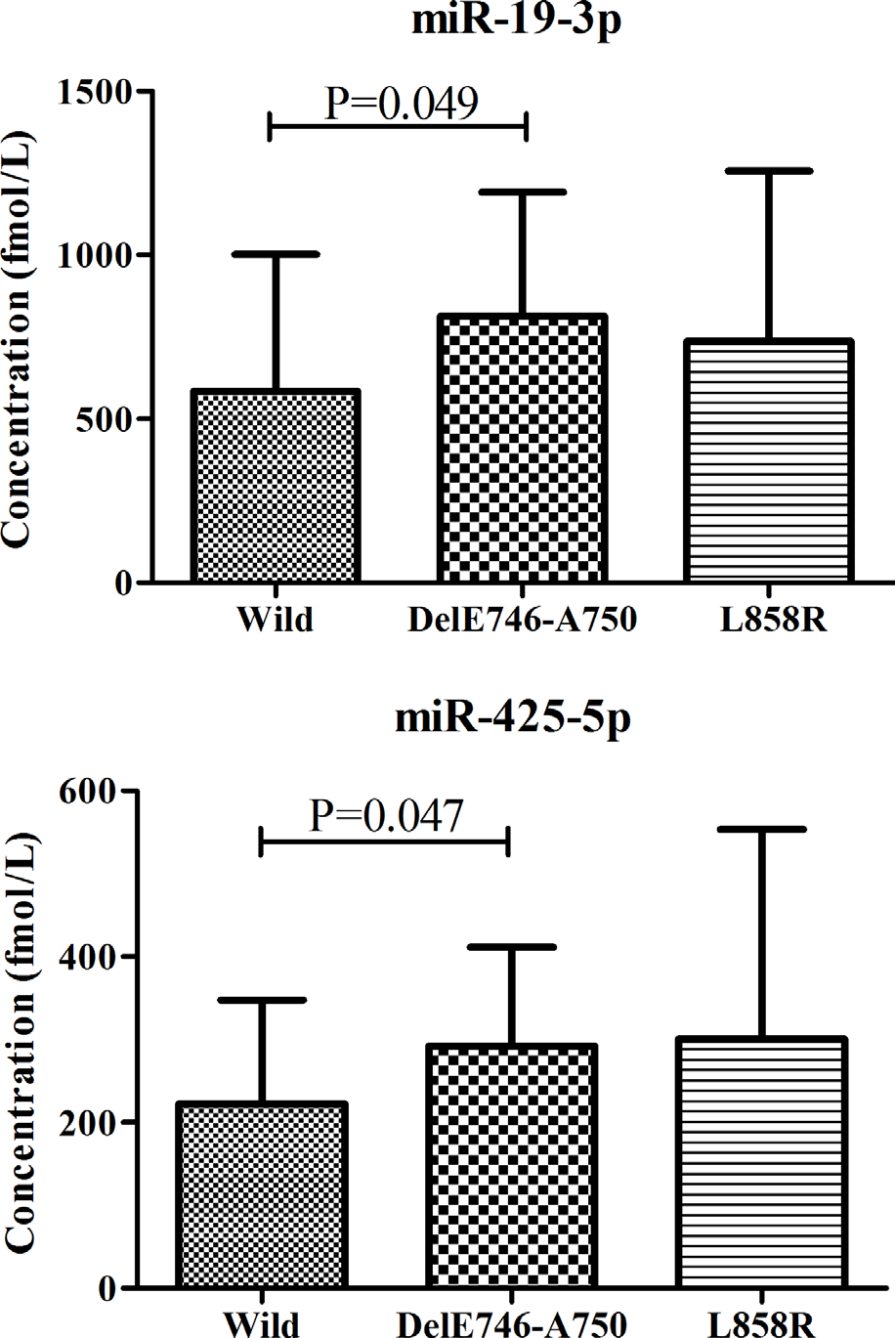
Different expression levels of miR-19-3p and miR-425-5p in peripheral plasma of LA patients according to EGFR status LA: lung adenocarcinoma.

To explore the relationship of the six-miRNA panel with clinical parameters, we analyzed data of all 141 LA patients. No miRNA or RSF was significantly related with gender, age, smoking status or tumor stage (*p*-values > 0.05). Epidermal growth factor receptor (EGFR) mutation status was examined in 46 patients in our study as part of routine pathology to guide decision-making regarding adjuvant therapy in the clinical (20 cases with wild type, 12 with DelE746-A750 mutation and 14 with L858R mutation). No significant difference of the six-miRNA panel was found in 20 patients with wild type and 26 cases with EGFR mutation. However, we found that plasma miR-19b-3p and miR-425-5p were significantly up-regulated in 12 patients with DelE746-A750 mutation compared to those with wild type (Figure 4).

### Evaluation of miRNAs in tissue samples

**Figure 5:**
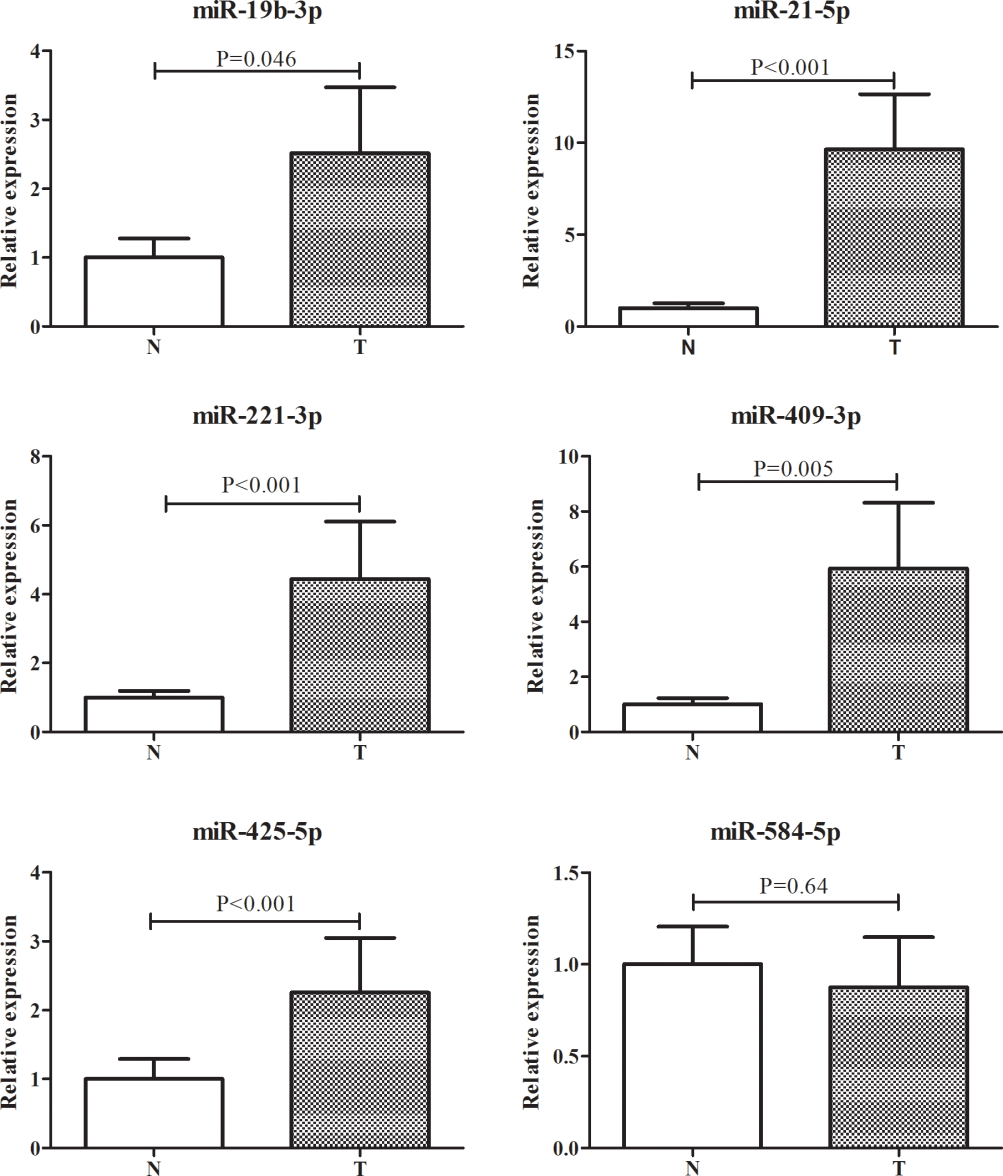
Expression of the six miRNAs in the tumor tissues of LA patients Y axis was presented as relative expression (normalized to *U6*; 2^−ΔΔCt^). Error bar: standard error. LA: lung adenocarcinoma; N: normal tissues; T: tumor.

Next, we examined the expression levels of the six miRNAs in an additional 19 pairs of tissue samples to evaluate the relationship of the identified miRNAs in peripheral plasma and tissues of LA patients. As shown in Figure 5, all the six miRNAs except miR-584-5p had higher expression in tumor samples than in normal tissues.

### Comparison of miRNAs in peripheral and arterial plasma

**Figure 6:**
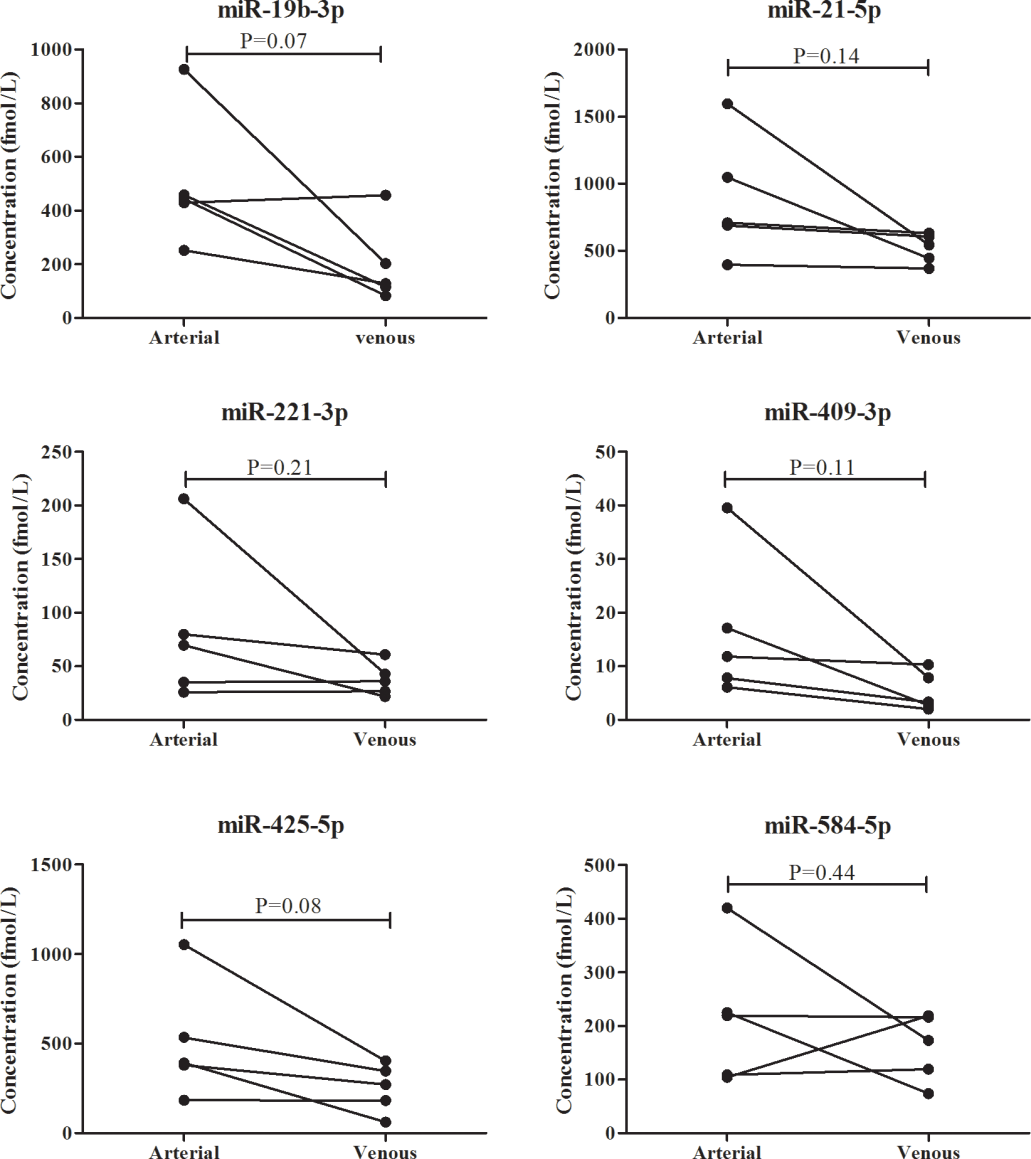
Comparison of the six miRNAs in 5 arterial plasma samples and matched peripheral plasma samples

As blood flows from arterial to venous circulation, we assessed expression levels of the miRNAs in 5 arterial plasma samples and compared those in the matched peripheral plasma samples from the same individuals. Among the six miRNAs, miR-19-3p, miR-21-5p, miR-221-3p, miR-409-3p and miR-425-5p showed higher expression levels in arterial plasma in more than a half of the subjects. However, due to the relatively small sample size, the results were not statistically significant (Figure 6).

### Identification of miRNAs in peripheral plasma exosomes

In addition, the expression levels of miRNAs in exosomes from 18 LA and 14 HC peripheral plasma samples were explored to assess the potential form of the identified miRNAs in peripheral plasma. All the six miRNAs were up-regulated but only miR-19-3p, miR-21-5p and miR-221-3p were with statistical significance in exosomes from LA plasma samples (Table 3).

**Table 3:** Expression levels of the six miRNAs in the peripheral exosomes of 18 LA patients and 14 HCs (presented as mean ± SD; 2^−ΔΔC^^t^)

miRNA	Cases	Controls	FC	*P* value
miR-19b-3p	0.79 ± 0.85	1.05 ± 0.32	1.3	0.02
miR-21-5p	0.49 ± 0.73	1.2 ± 0.82	2.46	< 0.001
miR-221-3p	0.39 ± 0.24	1.17 ± 0.65	3.02	< 0.001
miR-409-3p	0.81 ± 1.44	1.29 ± 1.61	1.59	0.111
miR-425-5p	0.98 ± 1.05	1.37 ± 1.25	1.39	0.129
miR-584-5p	0.62 ± 0.69	1.06 ± 0.62	1.69	0.063

FC: fold change.

## DISCUSSION

Majority of previous studies investigated the diagnostic performance of circulating miRNAs in NSCLC which comprised a range of subgroups with molecular and histological heterogeneity [15]. In the present study, we focused on LA and designed a four-phase study to identify peripheral plasma miRNAs which might have potential in detecting the disease. In the screening phase, differential expression profiling of plasma miRNAs were initially analyzed in 3 LA and 1 HC pooled samples with Exiqon miRNA qPCR panels which appeared to show better sensitivity and linearity than TaqMan platform while less abundant miRNAs were measured [22]. To control the false positive rate, miRNAs identified from Exiqon panels were confirmed in the same 40 plasma samples by qRT-PCR. In the following validation stages, the expression levels of plasma miRNAs were assessed by the method of absolute concentration analysis which might be the optimal way to normalize miRNA in body fluid samples. Through the training and the testing stage, a miRNA panel including six up-regulated peripheral plasma miRNAs (miR-19b-3p, miR-21-5p, miR-221-3p, miR-409-3p, miR-425-5p and miR-584-5p) was identified and could be used as a biomarker in diagnosis of LA. The external validation stage further verified the reliability of the diagnostic value of the six-miRNA panel. The six miRNAs were also explored in LA tissues. However, miR-19b-3p, miR-21-5p, miR-221-3p, miR-409-3p and miR-425-5p but not miR-584-5p showed consistently high expression in LA tissues.

Among the six miRNAs identified in our study, miR-19b-3p was one of the key oncogenic components of the miR-17-92 cluster. High miR-19b-3p expression in NSCLC tissues was related with higher TNM stage, lymph node metastasis and poorer survival. Enforced expression of miR-19b-3p could trigger epithelial-mesenchymal transition (EMT), enhance migration and invasion of lung cancer cells [23]. In addition, miR-19b-3p was also reported to be up-regulated in serum and served as an unfavorable predictor in prognosis of NSCLC [24]. As a well known onco-miRNA, miR-21-5p was studied thoroughly in various cancers including lung cancer [25, 26]. High expression of miR-21-5p was found in tissue samples, blood samples and sputum samples and could be used as a predictor in diagnosis, prognosis and monitoring therapy effect for patients with NSCLC [27–32]. Recently, TCGA project focused on comprehensive, multiplatform analysis of LA, with attention towards pathobiology and clinically actionable events which might be beneficial to further investigations of lung cancer [17]. They also found miR-21-5p was one of the 32 most discriminatory miRNAs between the matched LA/adjacent normal samples These results were consistent with our findings. As for miR-221-3p, controversial roles were found in the development and progression of lung cancer [33–35]. Heegaard et al. demonstrated that miR-221-3p was down-regulated in the serum but not in plasma sample of NSCLC patients [36]. But in our study, we found miR-221-3p was consistently up-regulated in plasma and tissues from LA patients. Thus, the conflicting function of miR-221-3p is needed to be further investigated. Though not studied in LA, miR-425-5p was found to be up-regulated and closely related to tumor stages and sizes in SCC tissues. It was also suggested that miR-425-5p might be a driver for tumor formation, growth, and progression to higher staging in SCC [37]. It was in accordance with our finding of miR-425-5p in LA. The other two miRNAs (miR-409-3p and miR-584-5p) were not explored in lung cancer and had distinct roles in different types of cancer [38–41]. In our study, miR-409-3p was up-regulated in both plasma and tissue samples from LA patients and might play an important role in LA. For miR-584-5p, we found that it was up-regulated in LA plasma but did not show differential expression level between LA and normal tissues. According to the theory that circulating miRNAs are originated from cells, we might assume that up-regulated plasma miR-584-5p might be a passenger but not a driver factor for tumorgenesis in LA. Future research of these miRNAs in LA formation and development is warranted.

The diagnostic capacity of the miRNAs identified in our study was also explored in some other cancers. Down-regulation of miR-19b-3p was found in plasma of gastric cancer and could distinguish normal population from patients with different TNM stages and grades [42]. Circulating miR-21 was also assessed to be a diagnostic biomarker in various cancers such as hepatocelluar carcinoma, gastric cancer and so on [43, 44]. Up-regulation of miR-221-3p in the circulation could also aid in the detection of gastric cancer, colorectal cancer, malignant melanoma, larynx cancer and pancreatic cancer [45–49]. To better take advantage of these miRNAs as biomarkers for specific cancer, future study is necessary.

As a member of the ErbB family of transmembrane receptor tyrosine kinases, EGFR is involved in signal transduction pathways that regulate apoptosis and proliferation [50]. NSCLC patients with activating mutations in the EGFR gene could benefit from the treatment with EGFR tyrosine kinase inhibitors (EGFR-TKI). Thus, EGFR mutation status of NSCLC patients is determined in the clinical to guide decision-making regarding adjuvant treatment [51]. Nowadays, sequencing remains to be the gold standard for EGFR mutation analysis, but the positive rate is low. EGFR mutation could also be detected by PCR technology (amplification refractory mutation system, ARMS) which is simple and convenient but with some non-specific reactions and relatively high false positive rate [52]. To better understand the complicated mechanism and biology of lung cancer, many studies have explored the association of miRNAs and EGFR in lung cancer [53, 54]. Some studies also identified some circulating miRNAs as potential substitutes for EGFR mutation and biomarkers for monitoring EGFR-TKI treatment [52, 55]. In our study, we found that plasma miR-19b-3p and miR-425-5p were significantly up-regulated in patients with DelE746-A750 mutation compared to those with wild type. Though without statistical significance, expression levels of the two plasma miRNAs were also higher in patients with L858R mutation compared to those with wild type. We thought that patients with high expression of plasma miR-19b-3p and miR-425-5p might be more sensitive to the EGFR-TKI treatment. However, due to the limited data, we did not explore the indicative role of the two miRNAs in monitoring the treatment effect of EGFR-TKI and the potentially prognostic value for LA patients in our study. Future study with more patients and comprehensive data is needed to validate our findings for the potential application of the miRNAs in the future clinical.

Derived from pulmonary circulation, arterial blood flows to systemic circulation and becomes venous plasma. Thus, we assumed that circulating miRNAs released from tumor cells might present higher expression levels in arterial blood than those in peripheral plasma. Our study revealed that miR-19b-3p, miR-21-5p, miR-221-3p, miR-409-3p and miR-425-5p but not miR-584-5p in LA plasma might be released from LA cells since the consistent expression tendency between tissue and plasma samples. Interestingly, comparison of the miRNAs in peripheral and arterial plasma showed that the other five miRNAs except miR-584-5p were up-regulated in arterial plasma in more than a half of the subjects. Although no result achieved statistical significance (miR-19-3p, miR-21-5p, miR-409-3p and miR-425-5p were borderline significant) potentially due to the small sample size, our findings could support the hypothesis in some extent.

Exosomes are small (40–100 nm) membrane vesicles secreted by most types of cell including cancer cell. Theses vesicles could protect the miRNAs, mRNAs and proteins from degradation by various enzymes outside and transfer messages among cells that make them the most important intercellular communicators [56]. Thus, the expression of the miRNAs identified in our study was also explored in exosomes to better understand the potential form of the miRNAs in peripheral plasma and utilize the potentially powerful diagnostic and therapeutic tool in the future. Our results showed that miR-19-3p, miR-21-5p and miR-221-3p were significantly up-regulated in peripheral plasma exosomes from LA patients. Reportedly, high level of exosomal miR-21 could act as diagnostic and/or prognostic biomarker in some cancers [57, 58]. The findings were consistent with our results. These three miRNAs might participate in the tumorgenesis and development of LA by exosomes and are worthy of further investigation. However, the other three miRNAs (miR-409-3p, miR-425-5p and miR-584-5p) did not show different expression between plasma exosomes from LA patients and HCs. It was reported that miRNAs were not only packaged into exosomes, they could also binding with proteins, such as the Argonaute 2 protein in the circulation [59]. Thus, we assumed that the majority of .miR-409-3p, miR-425-5p and miR-584-5p might bind to proteins in plasma of LA patients. The phenomena and the mechanism are warranted to be explored and validated in the future.

Some limitation of the study should be considered. First, Asian and Caucasian patients might have different molecular alterations, biological behaviors and clinical outcomes. However, we could not explore the role of the miRNAs in more patients with diverse populations. Future study with more cases with diverse populations might help to reveal the effect of population on the findings. Second, information about EGFR status was valid in 46 plasma samples. Only two types of mutation were included in our study. Meanwhile, the association of the identified miRNAs in tissue samples with EGFR was not explored. In the future, the relationship of the miRNAs and EGFR is needed to be explored widely. Third, only 5 pairs of arterial and peripheral venous blood samples were used in our study. Future studies with larger sample size are needed to validate the results of the miRNAs in arterial and peripheral plasma. In addition, our study focused on LA which was one subgroup of NSCLC. The diagnostic value and relationship of the miRNAs in other subtypes of NSCLC should also be explored in the future.

In conclusion, we identified a six-miRNA panel for LA detection. Our work will serve as the basis of the application of circulating miRNAs in clinical for the detection of LA in the future at least for Asian population to a certain extent. More effort should be taken to explore the roles and mechanisms of the miRNAs identified in our study in LA.

## MATERIALS AND METHODS

### Study design, patients and samples

All the subjects were recruited at First Affiliated Hospital of Nanjing Medical University between 2012 and 2014. All of the LA patients were histopathologically confirmed, and the tumor stage was determined according to the International Union Against Cancer's (UIAC) tumor-node-metastasis (TNM) system. Clinical characteristics for each patient were also recorded. And HCs enrolled in our study were healthy volunteers who conducted routine physical examination at First Affiliated Hospital of Nanjing Medical University

5 ml of blood sample from HCs and LA patients before initial treatment were collected with ethylenediaminetetraacetic acid (EDTA)-containing tubes (Becton,Dickinson and Company). The plasma was separated from blood within 12 hours after sample collection using a two-step protocol (350 RCF (reactive centrifugal force) for 10 min, 20,000 RCF for 10 min (Beckman Coulter, USA)) and then stored at −80°C for further processing. Tissue specimens were collected from surgery patients without preoperative chemoradiotherapy and kept in liquid nitrogen. The study was approved by Institutional Review Boards of the First Affiliated Hospital of Nanjing Medical University, and the written informed consent was obtained from each participant. The study was conducted according to the approved guidelines by the Hospital Ethics Committee.

Overall, a total of 141 LA patients and 124 HCs were enrolled in our study. The flow chart for the experiment design was shown in Figure 1. In the screening phase, Exiqon miRCURY-Ready-to-Use PCR-Human-panel-I+II-V1.M (Exiqon miRNA qPCR panel, Vedbaek, Denmark) was applied to identify differentially expressed miRNAs between 3 LA pool samples and 1 HC pool sample (30 peripheral plasma samples from LA patients and 10 from HCs were randomly selected and per 10 samples were pooled as 1 pool sample). The process of arrays and analyses for the differential miRNA profiling in LA plasma was in accordance with the previous study [60]. Briefly, 25 ng RNA isolated from each pool sample was reverse transcribed and further subjected to the Exiqon panels to detect the 168 miRNAs with relatively high abundance in plasma/serum. The relative expression between LA patients and HCs was assessed by 2^−ΔΔCt^ method, and ΔCt = average Ct (assay) – average Ct (normalizer assays).

After that, to verify the reliability and reproducibility of the results from arrays, the dysregulated miRNAs were confirmed in the identical 40 samples by qRT-PCR using the 2^−ΔΔCt^ method relative to cel-miR-39. In the training stage, the miRNAs discovered via the screening phase were determined in 42 LA samples and 32 HCs using qRT-PCR based absolute quantification methods. In the testing stage, the miRNAs identified in the training stage were further validated in plasma samples of 66 LA patients and 62 HCs. In the external validation stage, a total of 63 subjects including 33 cases and 30 controls were enrolled to further assess the diagnostic value of the identified miRNAs in LA.

An additional of nineteen pairs of LA tissue specimens and matched normal tissues were used to evaluate the expression level of identified miRNAs in tissue samples. Arterial blood samples were collected from 5 LA patients and were identified to compare the difference of miRNAs between peripheral and arterial plasma. Exosomal miRNAs were further assessed in 18 LA patients and 14 HCs.

### Isolation of exosomes

Exosomes from peripheral plasma were extracted using ExoQuick (System Biosciences, Mountain View, Calif) according to the manufacturer's protocol. Briefly, exosome pellets were precipitated from 100 μl ExoQuick exosome precipitation solutions and 400 μl plasma and then lysed in 200 μl RNase-free water for RNA extraction.

### RNA extraction

Total RNA from 200 μl plasma or exosomes was isolated using the mirVana PARIS Kit (Ambion, Austin, TX, USA) in accordance with the protocol. After the addition of denaturing solution (Ambion, Austin, TX, USA), 5 μl synthetic C.elegans miR-39 (5 nM/L, RiboBio, Guangzhou, China) was spiked into each sample for normalization of variation between samples. According to the manufacturer's instructions, Trizol (Invitrogen, Carlsbad, CA, USA) was used to extract total RNA from tissue samples. Total RNA was lysed in 100 μl RNase-free water and stored at −80°C for further analysis. The concentration and purity of the total RNA was analyzed using the ultraviolet spectrophotometer.

### Quantitative reverse transcription polymerase chain reaction

Bulge-Loop™ miRNA qRT-PCR Primer Set (RiboBio, Guangzhou, China) including specific primers for reverse transcription (RT) and polymerase chain reaction (PCR) was used to amplify miRNAs. The expression levels of miRNAs were determined by evaluating the level of fluorescence emitted by SYBR Green (SYBR^®^ Premix Ex Taq^™^ II, TaKaRa, Dalian, China). RT and PCR were conducted as previously described [60–62]. Briefly, RT reactions were carried out at 42°C for 60 min followed by 70°C for 10 min. The qRT-PCR was performed at 95°C for 20 sec, followed by 40 cycles of 95°C for 10 sec, 60°C for 20 sec and then 70°C for 10 sec on 7900HT real-time PCR system (Applied Biosystems, Foster City, CA, USA). The specificity of PCR products was evaluated by the melting curve analysis. The expression level of plasma miRNAs evaluated in the training, testing and external validation stage was assessed as absolute concentration based on a standard curve constructed with the use of synthetic miRNAs (micrON miRNA mimic, RiboBio, Guangzhou, China) [61]. The expression of miRNAs from tissue samples and exosomes was determined using the 2^−ΔΔCt^ method relative to RNU6B (U6) and the combination of spiked in cel-miR-39 with miR-16.

### Statistical analysis

The different expression levels of miRNAs were compared using paired *t*-test in paired arterial plasma and peripheral plasma samples and Mann-Whitney test in the other different groups. Clinical characteristics among different groups and their association with miRNA were evaluated with one-way ANOVA or χ^2^ test. The risk score of the identified miRNAs for LA was calculated [60, 63]. The receiver operating characteristic (ROC) curves were applied to evaluate the optimal cutoff value for each miRNA. The score (S) for each miRNA was set as 1 if the expression level of the miRNA was greater than the cutoff value, otherwise as 0. The risk score function (RSF) for subject i was:

Here, S_ij_ is the risk score of miRNA *j* on subject *i* and weighted by the regression coefficient (W_j_) estimated from univariate logistic regression models for each miRNA. The area under the ROC curves (AUC) was used to investigate the diagnostic value of the miRNAs for LA.

All the statistical analyses were performed with the use of SPSS16.0 software (SPSS Inc., Chicago, IL, USA). A two-sided *P* value < 0.05 was defined as statistical significance.

## SUPPLEMENTARY MATERIALS FIGURES AND TABLES


