# Supplementary material for: A six-microRNA panel in plasma was identified as a potential biomarker for lung adenocarcinoma diagnosis
Source: Oncotarget. 2016 Dec 27;8(4):6513–25. doi: 10.18632/oncotarget.14311 (PMC5351649; doi:10.18632/oncotarget.14311)
Supplement: Supplementary file 1 [file oncotarget-08-6513-s001.pdf]

A six-microRNA panel in plasma was identified as a potential biomarker for lung adenocarcinoma diagnosis

Supplementary Materials

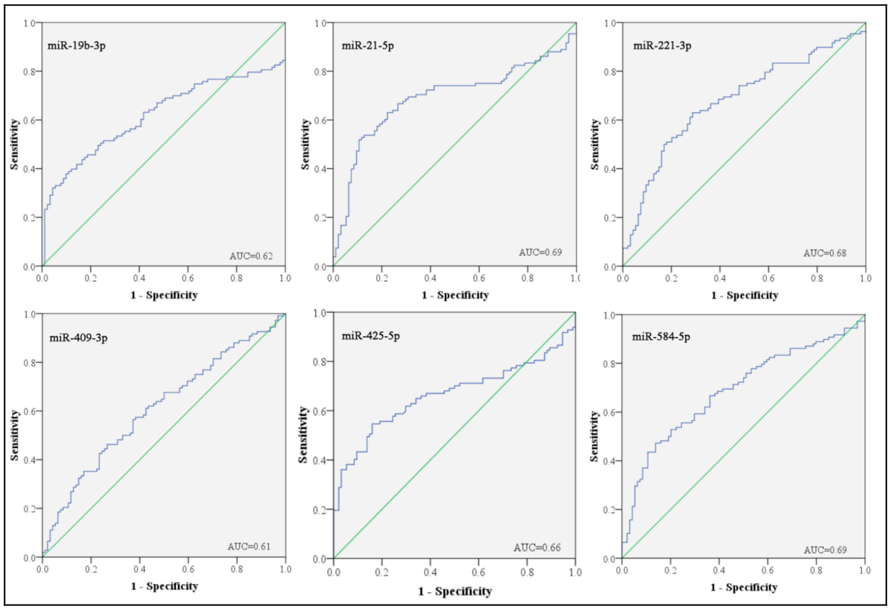

AUC: areas under the curve; LA: lung adenocarcinoma; HC: healthy control.

Supplementary Figure S1: Receiver-operating characteristic (ROC) curves of the six miRNAs to discriminate LA patients from HCs in the combined two cohorts of the training and the testing stage.

Supplementary Table S1: Differently expressed miRNAs in the screening phase

miRNA	FC (Exiqon panels)				Screen by qRT-PCR			
	Pool 1	Pool 2	Pool 3	Mean fold	T	H	FC	*P* value
miR-127-3p	4.13	2.10	2.89	3.04	−5.28 ± 1.06	−5.46 ± 0.37	0.88	0.94
miR-136-5p	4.83	2.42	2.11	3.12	8.11 ± 0.49	8.37 ± 1.59	1.20	0.66
miR-140-5p	3.41	2.41	2.64	2.82	7.18 ± 1.1	7.48 ± 0.61	1.23	0.32
miR-141-3p	2.28	3.97	4.43	3.56	8.4 ± 1.59	8.8 ± 1.8	1.32	0.57
miR-17-5p	3.18	4.82	2.98	3.66	4.62 ± 0.93	4.64 ± 0.35	1.01	0.86
miR-185-5p	2.06	2.29	2.16	2.17	4.25 ± 1.14	4.57 ± 0.63	1.16	0.58
miR-186-5p	2.47	3.74	2.21	2.80	8.72 ± 1.17	8.3 ± 0.67	0.75	0.21
miR-18a-5p	2.26	3.43	3.59	3.09	7.41 ± 0.8	6.98 ± 0.98	0.74	0.14
miR-18b-5p	2.37	3.60	4.71	3.56	7.66 ± 1.32	7.7 ± 0.62	1.03	0.97
miR-199a-3p	4.97	7.54	4.98	5.83	5.3 ± 0.78	4.93 ± 0.34	0.77	0.17
miR-199a-5p	4.36	2.48	4.31	3.72	8.47 ± 1.44	9.04 ± 0.57	1.48	0.46
miR-19a-3p	2.41	3.92	3.89	3.41	3.25 ± 1.6	3.13 ± 1.04	0.92	0.94
miR-19b-3p	2.66	2.10	2.17	2.31	3.72 ± 1.32	4.76 ± 1.07	2.06	**0.004**
miR-205-5p	2.92	2.44	4.31	3.22	7.21 ± 0.89	7.57 ± 0.84	1.28	0.49
miR-20a-3p	2.71	2.28	4.10	3.03	1.85 ± 1.27	1.3 ± 1.08	0.68	0.21
miR-20a-5p	2.00	3.01	2.23	2.42	6.37 ± 0.81	7.06 ± 0.65	1.61	**0.028**
miR-20b-5p	2.55	2.17	2.13	2.29	6.78 ± 1.8	7.5 ± 1.11	1.65	**0.039**
miR-21-5p	5.23	2.17	2.37	3.25	5.07 ± 0.91	5.91 ± 0.48	1.79	**0.001**
miR-210	3.04	4.61	2.33	3.33	7.31 ± 1.05	7.57 ± 1.37	1.20	0.39
miR-221-3p	3.37	5.11	2.75	3.74	3.99 ± 0.85	4.63 ± 0.64	1.56	**0.037**
miR-25-3p	2.05	2.84	3.23	2.71	2.62 ± 1.28	3.78 ± 1.16	2.23	**0.031**
miR-28-3p	5.42	8.22	2.50	5.38	8.09 ± 0.86	8.68 ± 0.57	1.51	**0.049**
miR-28-5p	2.09	4.36	2.28	2.91	8.53 ± 1.2	8.06 ± 0.92	0.72	0.29
miR-29b-2-5p	9.97	2.07	2.85	4.96	7.54 ± 1.45	9.04 ± 0.89	2.83	**0.024**
miR-29b-3p	2.10	2.36	3.18	2.55	8.34 ± 0.98	8.17 ± 0.85	0.89	0.91
miR-324-3p	2.24	2.20	2.16	2.20	6.08 ± 1.17	6.76 ± 0.86	1.60	**0.027**
miR-324-5p	5.39	2.48	8.17	5.35	7.43 ± 0.77	7.21 ± 0.52	0.86	0.53
miR-335-5p	5.57	8.45	2.63	5.55	7.65 ± 1.34	7.09 ± 1.06	0.68	0.14
miR-339-5p	3.42	4.88	7.32	5.21	2.48 ± 1.13	2.36 ± 0.81	0.92	0.69
miR-34a-5p	2.13	3.56	5.15	3.61	8.2 ± 0.67	7.72 ± 0.98	0.72	0.076
miR-382-5p	2.15	3.27	3.00	2.81	7.56 ± 1.32	7.66 ± 1.12	1.07	0.49
miR-409-3p	5.40	8.19	2.83	5.47	7.4 ± 1.27	8.49 ± 0.96	2.13	**0.033**
miR-425-5p	2.97	4.50	3.15	3.54	4.53 ± 1.11	5.2 ± 0.84	1.59	**0.012**
miR-543	3.00	4.56	3.13	3.56	8.2 ± 1.23	7.95 ± 1.17	0.84	0.36
miR-584-5p	3.98	6.04	3.48	4.50	6.93 ± 1.12	7.58 ± 1.12	1.57	**0.045**
miR-93-3p	2.08	3.16	4.35	3.20	7.31 ± 1.53	8.13 ± 1.14	1.77	**0.026**
miR-143-3p	−2.86	−2.31	−2.48	−2.55	6.29 ± 1.18	5.68 ± 1.09	0.66	**0.048**
miR-326	−7.09	−3.00	−2.19	−4.09	8.01 ± 0.97	7.43 ± 0.71	0.67	0.098
miR-144-5p	−4.42	−2.58	−2.03	−3.01	9.39 ± 0.56	9.55 ± 0.41	1.12	0.63

FC: fold change; T: tumor; H: healthy control.

Supplementary Table S2: Expression levels of the identified miRNAs from screening phase but not passed through the training stage (presented as mean ± SD; fmol/L)

miRNA	Cases	Controls	FC	*P* value
miR-20a-5p	2804 ± 1968	2313 ± 1089	1.21	0.7
miR-20b-5p	612 ± 321	705 ± 435	0.87	0.441
miR-25-3p	1514 ± 1179	1327 ± 797	1.14	0.721
miR-28-3p	161 ± 103	141 ± 98	1.14	0.37
miR-29b-2-5p	3845 ± 2782	3547 ± 2284	1.08	0.931
miR-324-3p	22.7 ± 10.7	20.8 ± 13.4	1.09	0.207
miR-93-3p	13.1 ± 13.9	12.1 ± 10.9	1.09	0.977
miR-143-3p	647 ± 365	735 ± 532	0.88	0.825

FC: fold change.

Supplementary Table S3: Expression levels of the six miRNAs in the external cohort (presented as mean ± SD; fmol/L)

miRNA	Cases	Controls	FC	*P* value
miR-19b-3p	693 ± 816	333 ± 190	2.08	0.036
miR-21-5p	1259 ± 1744	424 ± 209	2.97	< 0.001
miR-221-3p	253 ± 406	71 ± 49	3.56	< 0.001
miR-409-3p	8.9 ± 15	2.5 ± 2.1	3.51	0.009
miR-425-5p	293 ± 409	151 ± 79	1.94	0.029
miR-584-5p	702 ± 818	276 ± 277	2.54	< 0.001

FC: fold change.
